# Nodule unique de la vulve: penser à l'hidradénome papillifère

**DOI:** 10.11604/pamj.2014.19.301.5687

**Published:** 2014-11-19

**Authors:** Inssaf Ramli, Badredine Hassam

**Affiliations:** 1 Service de Dermatologie et Vénérologie, CHU Ibn Sina, Université Mohammed V, Souissi, Rabat, Maroc

**Keywords:** Hidradénome papillifère, tumeur annexielle, vulve, papillary hidradenoma, adnexal tumor, vulva

## Image en medicine

L'hidradénome papillifère est une tumeur kystique bénigne de la glande apocrine. C'est une affection rare apparaissant chez la femme de 20 à 89 ans au niveau des grandes lèvres et des régions périnéales et péri-anales. Cliniquement, il s'agit d'une papule ou nodule bien limité élastique, de couleur chaire, rose ou bleutée, parfois végétant et hémorragique ou pédiculé. Une association à une maladie de Paget extra-mammaire ou un adénocarcinome invasif a été décrite; ce qui impose une étude histologique permettant de confirmer le diagnostic, de rechercher les éventuelles pathologies associées et d’éliminer les autres diagnostics différentiels à savoir un kyste wolfien, une glande mammaire aberrante ou un carcinome épidermoïde. Le traitement de choix est l'exérèse chirurgicale. Nous rapportons le cas d'une patiente de 65 ans, sans antécédents notables, qui a présenté depuis 8 mois un nodule vulvaire indolore, mesurant 1 cm de diamètre, mobile, de couleur rose. L'histologie a confirmé le diagnostic d'un hidradénome papillifère en objectivant une prolifération bien limitée, sous forme d'une cavité avec de nombreuses lumières anastomosées, bordées par une couche de cellules cylindriques montrant une sécrétion apocrine apicale. Le diagnostic d'hidradénome papillifère a été retenu. La patiente a bénéficié d'une exérèse complète de la tumeur. Après deux ans de suivi, la patiente n'a pas présenté de récidive.

**Figure 1:**
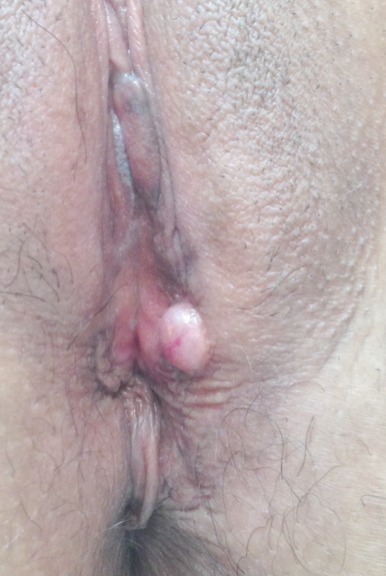
Nodule élastique et mobile à localisation vulvaire

